# Penetrance of Parkinson’s disease in *GBA1* carriers depends on variant severity and polygenic background

**DOI:** 10.1038/s41531-025-00997-y

**Published:** 2025-06-12

**Authors:** Emadeldin Hassanin, Zied Landoulsi, Sinthuja Pachchek, NCER-PD Consortium, Sinthuja Pachchek, Geeta Acharya, Gloria Aguayo, Myriam Alexandre, Muhammad Ali, Wim Ammerlann, Giuseppe Arena, Michele Bassis, Roxane Batutu, Katy Beaumont, Sibylle Béchet, Guy Berchem, Alexandre Bisdorff, Ibrahim Boussaad, David Bouvier, Lorieza Castillo, Gessica Contesotto, Nancy De Bremaeker, Brian Dewitt, Nico Diederich, Rene Dondelinger, Nancy E. Ramia, Angelo Ferrari, Katrin Frauenknecht, Joëlle Fritz, Carlos Gamio, Manon Gantenbein, Piotr Gawron, Laura Georges, Soumyabrata Ghosh, Marijus Giraitis, Enrico Glaab, Martine Goergen, Elisa Gómez De Lope, Jérôme Graas, Mariella Graziano, Valentin Groues, Anne Grünewald, Gaël Hammot, Anne-Marie Hanff, Linda Hansen, Michael Heneka, Estelle Henry, Margaux Henry, Sylvia Herbrink, Sascha Herzinger, Alexander Hundt, Nadine Jacoby, Sonja Jónsdóttir, Jochen Klucken, Olga Kofanova, Pauline Lambert, Zied Landoulsi, Roseline Lentz, Laura Longhino, Ana Festas Lopes, Victoria Lorentz, Tainá M. Marques, Guilherme Marques, Patricia Martins Conde, Deborah Mcintyre, Chouaib Mediouni, Francoise Meisch, Alexia Mendibide, Myriam Menster, Maura Minelli, Michel Mittelbronn, Saïda Mtimet, Maeva Munsch, Romain Nati, Ulf Nehrbass, Sarah Nickels, Beatrice Nicolai, Jean-Paul Nicolay, Fozia Noor, Clarissa P. C. Gomes, Claire Pauly, Laure Pauly, Lukas Pavelka, Magali Perquin, Achilleas Pexaras, Armin Rauschenberger, Rajesh Rawal, Lucie Remark, Ilsé Richard, Olivia Roland, Kirsten Roomp, Eduardo Rosales, Stefano Sapienza, Venkata Satagopam, Sabine Schmitz, Reinhard Schneider, Jens Schwamborn, Raquel Severino, Amir Sharify, Ruxandra Soare, Ekaterina Soboleva, Kate Sokolowska, Maud Theresine, Hermann Thien, Elodie Thiry, Rebecca Ting Jiin Loo, Johanna Trouet, Olena Tsurkalenko, Michel Vaillant, Carlos Vega, Liliana Vilas Boas, Paul Wilmes, Evi Wollscheid-Lengeling, Gelani Zelimkhanov, Rejko Krüger, Patrick May, Dheeraj Reddy Bobbili, Peter Krawitz, Carlo Maj, Rejko Krüger, Patrick May, Dheeraj Reddy Bobbili

**Affiliations:** 1 Luxembourg Centre for Systems Biomedicine, University of Luxembourg, Esch-Sur-Alzette, Luxembourg; 2 Institute for Genomic Statistics and Bioinformatics, University of Bonn, Bonn, Germany; 3 Transversal Translational Medicine, Luxembourg Institute of Health, Strassen, Luxembourg; 4 Centre for Human Genetics, University of Marburg, Marburg, Germany; 5 Department of Neurology, Centre Hospitalier de Luxembourg, Strassen, Luxembourg; 6 Luxembourg Institute of Health, Strassen, Luxembourg; 7 Centre Hospitalier de Luxembourg, Strassen, Luxembourg; 8 Laboratoire National de Santé, Dudelange, Luxembourg; 9 Association of Physiotherapists in Parkinson’s Disease Europe, Esch-sur-Alzette, Luxembourg; 10 Faculty of Science, Technology and Medicine, University of Luxembourg, Esch-sur-Alzette, Luxembourg; 11 Department of Epidemiology, CAPHRI School for Public Health and Primary Care, Maastricht University Medical Centre, Maastricht, the Netherlands; 12 Private practice, Ettelbruck, Luxembourg; 13 Parkinson Luxembourg Association, Leudelange, Luxembourg; 14 Luxembourg Center of Neuropathology, Dudelange, Luxembourg; 15 Department of Life Sciences and Medicine, University of Luxembourg, Esch-sur-Alzette, Luxembourg

**Keywords:** Genetics, Clinical genetics, Medical genetics, Population genetics, Parkinson's disease

## Abstract

Heterozygous *GBA1* variants increase Parkinson’s disease (PD) risk with variable penetrance. We investigated the interaction between genome-wide polygenic risk scores (PRS) and severity of pathogenic *GBA1* variants (*GBA1*_PVs_) to assess their combined impact on PD risk. *GBA1* variants were identified from whole exome sequencing in the UK Biobank and targeted PacBio sequencing in the Luxembourg Parkinson’s Study, with PRS calculated using genome-wide significant SNPs. *GBA1*_PVs_ were present in 8.8% of PD patients in the UK Biobank and 9.9% in LuxPark, with carriers showing consistently higher PD risk across all PRS categories. In the highest PRS category, PD risk increased 2.3-fold in the UK Biobank and 1.6-fold in LuxPark. Severe and mild *GBA1* variants conferred nearly double the risk of PD compared to risk variants. Our findings demonstrate the impact of PRS on *GBA1*_PVs_ penetrance, highlighting implications for genetic counseling and clinical trial design in *GBA1*-associated PD.

## Introduction

Pathogenic variants in the *GBA1* gene encoding the lysosomal enzyme glucocerebrosidase (GCase) are the most common genetic risk factors for Parkinson’s disease (PD)^1,2^. Initially associated with Gaucher’s disease (GD) in its biallelic form, *GBA1* variants also increase PD risk in heterozygous carriers. *GBA1* variants are prevalent in 5–20% of PD patients in different populations worldwide, with a recent study identifying a common intronic variant in 40% of African PD patients^3^. The risk of developing PD follows a gradient, with severe GD-causing variants (e.g., L483P) raising PD risk 9- to 10-fold, while mild variants (e.g., N409S) increase PD risk approximately 4-fold^2,4^. Additionally, non-GD-causing *GBA1* variants, such as E365K and T408M, are more frequently observed in PD patients and are considered significant risk factors for PD^2,4^. *GBA1*-associated PD (*GBA1*-PD) is typically characterized by earlier onset, more rapid progression, and a higher frequency of non-motor symptoms^5^.

The penetrance of *GBA1*-PD is variable and age-dependent, with estimates ranging from approximately 7.6% at 50 years to 29.7% at 80 years under a dominant model^6^. This gradual increase in risk complicates accurate risk assessment for carriers^7^. Therefore to define individual risk, it is essential to understand the role of a ‘genetic background’ as defined by common variants associated with PD risk and identified by genome-wide association studies (GWAS)^8^ among *GBA1* carriers, such as the polygenic background has been found to modulate the PD risk of *GBA1* variants in carriers of p.E365K, p.T408M and p.N409S variants, and decreasing the age of onset (AAO) of PD^9^. Moreover, variants in the PD genetic risk score were more frequent in GD patients who developed PD, suggesting that common variants may play a role in shared biological pathways underlying both conditions^10^.

Our study extends previous research by analyzing the impact of polygenic risk scores (PRS) across a broader range of *GBA1* pathogenic variant carriers (*GBA1*_PVs_) in the UK Biobank (UKB), with validation in the Luxembourg Parkinson’s Study (LuxPark), focusing on how PRS and *GBA1*_PV_ severity influence PD risk.

## Results

### Demographic characteristics and distribution of *GBA1* variants in UKB and LuxPark

After QC, the final UKB dataset included 185,225 individuals with available WES and genotyping data at the analysis time. Descriptive statistics of the study population after filtering are shown in Table 1. The dataset consisted of 1636 PD cases, with a mean AAO of 64.6 years, and 183,589 healthy control participants, with a mean age at assessment (AAA) of 64.1 years. In the LuxPark replication cohort, a total of 1430 individuals were included, comprising 653 PD cases with a mean AAO of 62.4 years and 767 healthy controls with a mean AAA of 59.6 years. The LuxPark cohort exhibited a significantly higher proportion of individuals with a positive family history of PD (FH + ) compared to the UKB cohort (chi-squared test *P* < 0.01). In UKB, 57 *GBA1*_PVs_ were identified in 9019 (4.8%) controls and 145 PD cases (8.8%). The mean of AAO for *GBA1*_PVs_ carriers was 66.2 ± 7.2 years for PD patients and the mean of AAA was 56.6 ± 7.97 for healthy controls. Out of the 57 *GBA1*_PVs_, 40 were classified as severe, 14 as mild, and three as risk variants, with risk variants being the most common (*n* = 7990 carriers, Table 2). In the LuxPark cohort, targeted PacBio sequencing of the *GBA1* gene previously revealed that 12.1% (77/637) of PD patients carried *GBA1* variants, with 10.5% (67/637) harboring known pathogenic variants, including severe, mild, and risk variants^11^. For this study, we focused on a subset of individuals who were both genotyped and *GBA1* PacBio-sequenced, identifying 99 carriers of *GBA1*_PVs_. This subset comprised 65 PD cases (9.9%) and 34 controls (4.4%). The mean AAO for PD cases was 61.5 ± 11.7 years, while the mean AAA for healthy controls was 59.4 ± 13.2 years. Of the 12 *GBA1*_PVs_ detected, nine were classified as severe, one as mild, and two as risk variants, with risk variants being the most common (*n* = 68, Table 2).

**Table 1:** Characteristics of the UK Biobank cohort and the Luxembourg Parkinson’s Study

Cohort	UK Biobank		Luxembourg Parkinson's study	
**Diagnostic**	Cases	Controls	Cases	Controls
**Participants,** ***n***	1636	183,589	653	767
**Male,** ***n*** **(%)**	1009 (61.7)	82,276 (44.8)	440 (67.4)	410 (53.5)
**Female,*n*** **(%)**	627 (38.3)	101,313 (55.2)	213 (32.6)	357 (46.5)
**Age at Onset, mean (SD)**	64.6 (8.6)	–	62.4 (11.5)	–
**Age at assessment, mean (SD)**	62.7 (5.3)	64.1 (8.0)	67.3 (10.7)	59.6 (7.12)
***GBA1PVs carriers, n (%)***	145 (8.8)	8874 (4.8)	65 (9.9)	34 (4.4)
**Family history of PD,*n*** **(%)**	157 (9.6)	7799 (4.2)	186 (28.5)	253 (33.0)

**Table 2:** Pathogenic GBA1 variants used in this study

*GBA1* variants	RS_id	Protein_change	Severity	UK Biobank		Luxembourg Parkinson's study	
				PD	HC	PD	HC
1:155235002:C:T	rs75822236	p.R535H	mild		8		
1:155235205:C:T	rs369068553	p.V499M	mild		7		
1:155235843:T:C	rs76763715	p.N409S	mild	10	685	7	3
1:155236367:G:A	rs374306700	p.R368C	mild		6		
1:155236384:G:A	rs76539814	p.T362I	mild		5		
1:155237394:G:A	rs1264734195	p.R316C	mild		3		
1:155237411:C:T	rs74731340	p.S310N	mild		1		
1:155237412:T:C	rs1057942	p.S310G	mild		3		
1:155237576:A:T	rs74500255	p.F255Y	mild		13		
1:155238570:C:G	rs147138516	p.D179H	mild	2	33		
1:155238620:A:G	rs794727783	p.M162T	mild		1		
1:155239639:A:C	rs794727708	p.L144R	mild		5		
1:155239934:G:A	rs1141814	p.R87W	mild		1		
1:155235790:C:T	rs149171124	p.E427K	risk	1	73		
1:155236246:G:A	rs75548401	p.T408M	risk	37	2668	16	12
1:155236376:C:T	rs2230288	p.E365K	risk	79	4948	23	16
1:155235195:C:T	rs80356772	p.R502H	severe		1	1	
1:155235196:G:A	rs80356771	p.R502C	severe	4	141		
1:155235197:G:C		p.N501K	severe		3		
1:155235727:C:G	rs1064651	p.D448H	severe	1	23		
1:155235757:C:T		p.D438N	severe		1		
1:155235772:C:A	rs80356769	p.V433L	severe		1		
1:155235810:C:T		p.W420*	severe		2		
1:155235814:C:T		p.D419N	severe		2		
1:155235823:C:T	rs121908311	p.G416S	severe		16		
1:155236277:G:A	rs121908309	p.R398*	severe		6	1	
1:155236295:G:A		p.R392W	severe		2		
1:155236409:C:G	rs398123526	p.D354H	severe	1	11		
1:155237357:G:A	rs121908298	p.P328L	severe		1		
1:155237370:G:A	rs765633380	p.R324C	severe		5		
1:155237425:D:1		p.P305Lfs*30	severe		11		
1:155237444:A:G	rs794727908	p.I299T	severe		2		
1:155237453:C:T	rs78973108	p.R296Q	severe	1	45		
1:155237458:A:C	rs367968666	p.H294Q	severe	1	33		1
1:155238141:A:T	rs381737	p.F252I	severe		2	1	
1:155238174:C:T	rs409652	p.G241R	severe		6	2	
1:155238192:A:G	rs1064644	p.S235P	severe		1		
1:155238194:C:T	rs74462743	p.G234E	severe		3		
1:155238215:T:C	rs364897	p.N227S	severe	1	14		
1:155238228:A:G	rs61748906	p.W223R	severe	1	2		
1:155238234:G:T	rs866075757	p.P221T	severe		10		
1:155238260:G:C		p.S212*	severe	4	5		
1:155238270:G:A	rs398123532	p.R209C	severe		4		
1:155238291:G:A	rs1009850780	p.R202*	severe		4		
1:155238298:D:2	rs749714463	p.L199Dfs*61	severe	1			
1:155238519:T:G	rs121908297	p.K196Q	severe		7		
1:155238597:G:A	rs398123530	p.R170C	severe	1	6		
1:155238629:C:T	rs79653797	p.R159Q	severe		5		
1:155239633:G:A	rs758447515	p.S146L	severe		5		
1:155239656:D:1		p.P138Lfs*61	severe		2		
1:155239736:G:A		p.Q112*	severe		1		
1:155239989:I:1		p.T69Dfs*11	severe		3		
1:155240660:I:1	rs387906315	p.L29Afs*17	severe		3		
1:155241085:D:2	rs766291162	p.E9Gfs*7	severe		25		
1:155235252:T:C	rs421016	p.L483P	severe			10	1
1:155238195:G:T		p.G234W	severe			1	
1:155238624:C:T	rs121908299	p.P161S	severe			2	
1:155240629:G:A		c.115+1G>A	severe			1	1

Summary of the identified GBA1 variants, including missense, nonsense, and indel variants. Missense and nonsense variants are reported using the format CHR:POS:REF:ALT, where CHR represents the chromosome, POS is the genomic position (GRCh37/GRCh38), REF is the reference allele, and ALT is the alternate allele. Indels (insertions and deletions) follow a different format, CHR:POS:TYPE:SIZE, where CHR is the chromosome, POS is the genomic position, TYPE specifies the variant type (D for deletions and I for insertions), and SIZE indicates the number of nucleotides affected.

*PD* Parkinson’s disease, *HC* healthy controls.

### Combined effect of PRS and *GBA*_pvs_ status and severity on PD risk

We calculated the PRS using a panel of SNPs to investigate the influence of the genetic background on PD risk in PD *GBA1*_PVs_ carriers. Our analysis revealed that the PRS was significantly higher in PD patients compared to healthy controls in both cohorts (Wilcoxon test *P* < 0.01).

**Fig. 1:**
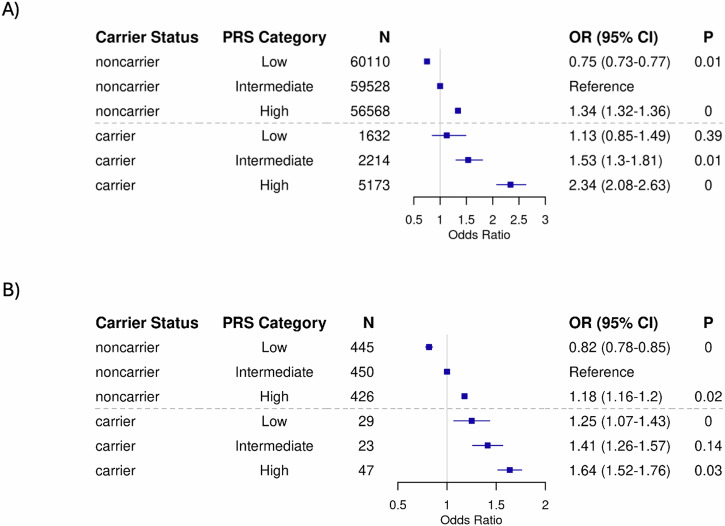
Parkinson’s disease (PD) risk stratified by *GBA1* carrier status and polygenic risk scores (PRS) categories. Odds ratio for PD were estimated using logistic models, while conditioning on the sex, age at assessment and the first four ancestry principal components in both UK Biobank (**A**) and the Luxembourg Parkinson’s Study (**B**). Non-carriers with intermediate PRS served as the reference group. Carriers and non-carriers were categorized into categories based on their PRS.

**Fig. 2:**
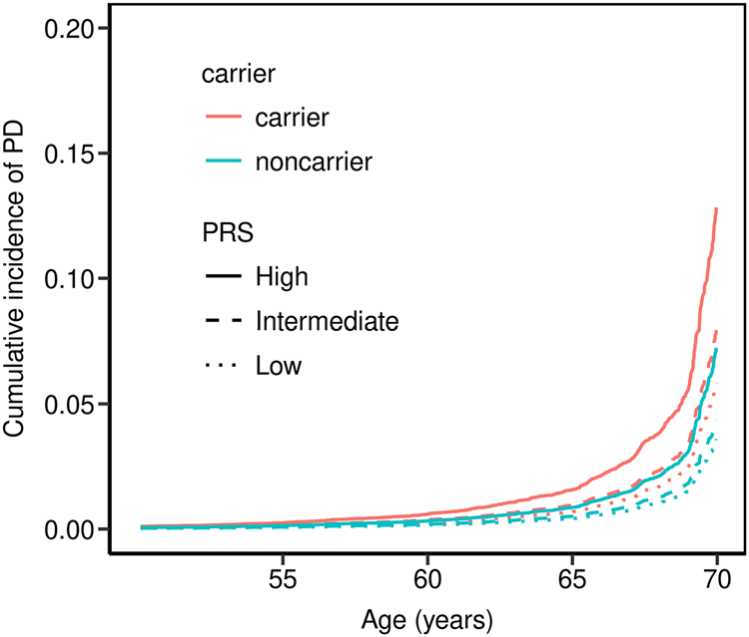
Cumulative incidence of Parkinson’s disease (PD) stratified by *GBA1* carrier status and polygenic risk scores categories in the UK Biobank. Cumulative incidence was estimated using Cox proportional hazards models, adjusted for sex and the first four ancestry principal components. Participants were stratified by GBA1 carrier status as well as by PRS categories, in the UK Biobank. Red lines indicate GBA1 carriers and green lines indicate non-carriers. Line styles represent PRS categories, with solid lines for high PRS, dashed lines for intermediate PRS, and dotted lines for low PRS.

We assessed the influence of PRS and *GBA1*_PVs_ carrier status on PD risk in both cohorts by calculating ORs for PD across PRS categories, using non-carriers with intermediate-PRS as the reference group. PD risk was consistently higher in *GBA1*_PVs_ carriers compared to non-carriers across all PRS categories in both cohorts (Fig. 1). In UKB, non-carriers with low- or high-PRS had PD ORs of 0.75 (0.73–0.77) or 1.34 (1.32-1.36) respectively (Fig. 1). Among GBA1_PVs_ carriers, those with high-PRS category exhibited OR of 2.34 (95% CI, 2.08–2.63) compared to carriers with low-PRS (OR: 1.13; 95% CI, 0.85–1.49) (Fig. 1). Similarly, in LuxPark, *GBA1*_PVs_ carriers with high-PRS had PD ORs of 1.67 (95% CI, 1.55–1.79) compared to those with low PRS (OR: 1.25; 95% CI, 1.07–1.43), although the effect was less pronounced than in UKB. No significant interaction between *GBA1*_PVs_ carrier status and PRS was observed in either UKB (*P* = 0.73) or LuxPark (*P* = 0.48). The Cox proportional hazards analysis confirmed the combined effect of carrier status and PRS on PD risk (Fig. 2, Supplementary Table 1). In UKB, among *GBA1*_PVs_ carriers, individuals with a high PRS had the highest cumulative incidence of PD, reaching 12.8% by the age of 70. In contrast, carriers with a low PRS had a lower cumulative incidence of approximately 5.8% at the same age. At the same age, the cumulative incidence of the disease is consistently higher in *GBA1*_PVs_ carriers compared to non-carriers. Similar PRS-stratified trends were observed in the LuxPark cohort; corresponding estimates are provided in the Supplementary Fig. 1. We performed a fisher test for each of 42 SNPS in cases compared to controls, to identify potential penetrance modifiers. After applying FDR correction for multiple testing, no SNPs showed significant differences in *GBA1* carriers. However, in non-carriers, eight SNPs were significantly enriched in PD patients (FDR-adjusted *p* value < 0.05). Among these, only rs34311866 was replicated in the LuxPark cohort. The full results are provided in Supplementary Table 2.

**Fig. 3:**
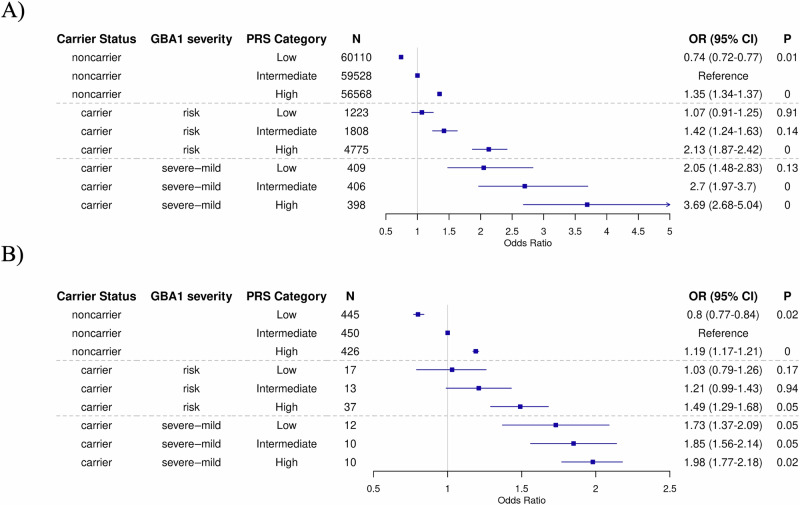
Parkinson’s disease (PD) risk stratified by severity status of *GBA1* carrier and polygenic risk score (PRS) categories. Odds ratio (OR) for PD were estimated using logistic models, while conditioning on the sex, age at assessment and the first four ancestry principal components in both UK Biobank (**A**) and the Luxembourg Parkinson’s Study (**B**). Non-carriers with intermediate PRS served as the reference group.

To gain a deeper understanding of how disease severity is influenced by PRS, we further examined the combined effect of PRS and *GBA1*_PVs_ severity, categorized in two groups: “severe+mild” and “risk”. In both cohorts, we found an association between the severity of *GBA1*_PVs_ and a higher OR for PD (Fig. 3). Carriers of severe+mild *GBA1*_PVs_ tended to have higher risk of PD (almost twofold or higher) compared to carriers of risk *GBA1*_PVs_, regardless of their PRS category. In UKB, ORs for carriers of “severe+mild” *GBA1*_PVs_ ranged from 2.05 (95% CI, 1.48–2.83) to 3.69 (95% CI, 2.68–5.04) across the PRS categories, while for carriers of risk *GBA1*_PVs,_ ORs ranged from 1.07 (95% CI, 0.91–1.25) to 2.13 (95% CI, 1.87–2.42, Fig. 3). Similarly, in LuxPark, ORs for carriers of “severe+mild” *GBA1*_PVs_ ranged from 1.73 (95% CI, 1.37–2.09) to 1.98 (95% CI, 1.77–2.18) across the PRS categories, while for carriers of risk *GBA1*_PVs,_ ORs ranged from 1.03 (95% CI, 0.79–1.26) to 1.49 (95% CI, 1.29–1.68, Fig. 3). No significant interaction between *GBA1*_PVs_ severity (respectively for severe+mild or risk) and PRS was observed in either UKB (*P* = 0.99 and *P* = 0.26) or LuxPark (*P* = 0.44 and *P* = 0.26). We performed an additional analysis grouping mild with risk variants (risk+mild) separately from severe variants. The results showed the same overall trend, with the odds of PD increasing when severe variants were analyzed independently, specifically in UK biobank (Supplementary Figs. 2a, 2b).

## Discussion

In this study, we investigated the combined effect of polygenic background and PD-associated *GBA1* variants on the risk of developing PD across two cohorts. Our findings show that both PRSs and *GBA1*_PVs_ independently contribute to PD risk. Individuals carrying *GBA1*_PVs_ consistently showed a higher baseline risk of developing PD compared to non-carriers, regardless of their PRS group. Notably, PRSs independently elevated the odds of developing PD in both carriers and non-carriers. These results suggest that the effects of *GBA1*_PVs_ and PRSs are additive, where the presence of *GBA1*_PVs_ contributes a fixed increase in baseline risk, and PRS independently further increases the overall risk of PD. At older ages, cumulative incidence of PD is consistently higher in *GBA1*_PVs_ compared to non-carriers, therefore, highlighting the increased risk associated with carrying *GBA1*_PVs_.

Among *GBA1*_PVs_ carriers, the cumulative incidence is higher in the high-PRS group compared to the low-PRs group, a trend also observed non-carriers. This suggests that PRS have an additive effect on the penetrance of *GBA1*_PVs_, amplifying the overall risk of PD. The earlier observed risk in the LuxPark cohort compared to the UKB likely results from differences in study design and recruitment strategies. The UKB represents a general population study, where early-onset PD is underrepresented, potentially leading to a later onset of PD. In contrast, LuxPark focuses on PD patients, including individuals with both early- and later-onset forms of the disease. Since such studies do not capture the full population at risk, clinically relevant cumulative incidence estimates are not suitable here; however, the data from LuxPark reflect a higher proportion of individuals with early-onset PD, leading to an earlier observed risk compared to the UKB.

There is notable variability in cumulative risk estimates for *GBA1* carriers across studies, as shown in Supplementary Table 3. For example, Rana et al.^11^ reported a 1.0% risk at age 60, Anheim et al.^6^ found a 21.4% risk at age 70 in familial cohorts, while Alcalay et al.^12^ reported risks in an Ashkenazi Jewish cohort, with 5.2% risk in *GBA1* carriers and 9.1% at age 70 in patients with Gaucher disease. In the UKB, we report a 1.7% risk in *GBA1* carriers at age 60 and 10.1% at age 70. This variability likely arises from differences in study design, and cohort characteristics. Furthermore, the variance observed across PRS categories, particularly in *GBA1* carriers, may contribute to these differences in risk estimates. PRS modulates this risk, as our results show a higher cumulative incidence at age 70 for *GBA1* carriers with high PRS (12.8%) compared to those with low PRS (5.8%), contributing to the observed variance in risk estimates.

Using the same approach, we investigated the specific effect of *GBA1*_PVs_ severity, classifying them into two groups: “severe+mild” and “risk” variants. Carriers of severe+mild *GBA1*_PVs_ exhibited the highest baseline risk for developing PD, followed by those with risk variants, further emphasizing the dose-dependent effect of *GBA1*_PVs_ on disease susceptibility. Importantly, PRS continued to elevate PD risk independently and additively, regardless of variants severity.

Our results suggest a clear gradient of risk driven by both *GBA1*_PVs_ severity and PRS. Individuals carrying severe *GBA1*_PVs_ in combination with a high PRS have the highest risk of developing PD, while those with risk variants and a low PRS have a lower, but still elevated, risk compared to non-carriers. Notably, non-carriers with a high PRS also have a substantial risk, but it remains lower than that of any variant carriers with similar PRS levels. These results highlight the complex interplay between polygenic risk and *GBA1*_PVs_, underscoring the importance of considering both factors in genetic assessments of PD risk.

Our findings are consistent with previous studies demonstrating that PRS not only modifies PD risk but also reduces the AAO in carriers of *GBA1* variants, specifically for the two risk variants p.E365K and p.T408M, and the mild p.N409S variant^9^. Additionally, variants included in the PD PRS were found to be more frequent in patients with GD type 1 who developed PD, suggesting that common genetic risk variants may influence shared underlying biological pathways^10^.

A key finding of this study is that *GBA1* variants and PRS independently and additively increase the risk of PD, with both factors separately contributing to the overall risk. This highlights the importance of considering PRS in clinical trial designs for *GBA1*-related PD, using pre-trial genetic analysis to stratify patients by both *GBA1* variants and overall genetic risk^9,13,14^. Incorporating PRS alongside whole-exome or targeted gene sequencing in initial diagnoses could streamline healthcare costs, expand cohort sizes, and support the integration of genetic risk data into routine clinical practice^15^. Furthermore, combining genetic information with non-genetic factors, such as family history and presymptomatic phenotypes, could improve disease prediction and promote the development of multifactorial risk models^16,17^.

The results of PRS studies can be influenced by factors such as study design and recruitment strategies^18^. In this study, the effects were more pronounced in the discovery cohort (UKB), a population-based sample, compared to the replication cohort (LuxPark), a PD-specific case-control cohort. While both cohorts showed similar trends, the differences are likely due to specific cohort characteristics and recruitment methods. Notably, a significant discrepancy in family history between PD cases and controls was observed in the UKB but not in the Luxembourg cohort, further emphasizing the impact of study design on the outcomes.

This study has several limitations. The UKB cohort is not PD-specific and lacks age and sex matching between cases and controls; however, we mitigated this by including age and sex as covariates in the regression analysis. Additionally, the sample size is limited in certain PRS or *GBA1*_PVs_ severity categories, which may lead to overestimation of effect sizes. These factors should be carefully considered when interpreting the findings. The LuxPark cohort is enriched for individuals with PD and includes relatively few *GBA1* carriers without PD. Consequently, it is not appropriate for estimating population-level age-specific risk or cumulative incidence. Cumulative risk estimates presented for LuxPark in the supplementary material should be interpreted with caution, as they reflect cohort-specific trends rather than generalizable risk estimates.

Overall, this study shows that both PRS and *GBA1*_PVs_ contribute independently and additively to the risk of PD. Carriers of *GBA1*_PVs_ consistently show a higher baseline risk, particularly those with severe variants and high PRS, with PRS influencing *GBA1*_PVs_ penetrance. These results highlight the need to integrate PRS and *GBA1*_PVs_ into PD genetic assessment for better risk stratification. By deepening our understanding of this genetic landscape, especially variant severity, we can pave the way for personalized therapeutic strategies. Ultimately, this approach will bring us closer to tailoring treatments to individual genetic profiles, optimizing outcomes and improving patients’ quality of life.

## Methods

### The UK biobank cohort

UKB is a large, long-term prospective study comprising over 500,000 participants^19^. For this study, we included 185,225 individuals (1636 PD patients and 183,589 healthy controls) of European ancestry with both genotyping and whole-exome sequencing (WES) data available. Participants were genotyped using the UKB Axiom Array, and imputation was performed with the Haplotype Reference Consortium and UK10K + 1000 Genomes reference panels. Whole-exome sequencing was conducted using the IDT xGen Exome Research Panel v1.0^20^. Ethics approval for the UK Biobank (UKB) study was obtained from the Northwest Multicentre for Research Ethics Committee (MREC). The UKB ethics statement is available at the following website (https://www.ukbiobank.ac.uk/learn-more-about-uk-biobank/about-us/ethics). All UKB participants provided informed consent at recruitment. PD diagnosis was based on self-reports by participants or the International Classification of Diseases (ICD-10) diagnosis codes. This included individuals with self-reported code 1262 or ICD-10 code of G20 in hospitalization records. Quality control (QC) followed standard procedures. We excluded outliers with putative sex chromosome aneuploidy (field 22019), high heterozygosity or missing genotype rates (field 22027), and discordant reported versus genotypic sex (field 22001). The analysis was restricted to unrelated individuals to the second degree. The dataset is available for research purposes, and all participants provided documented consent. UKB analyses were conducted using a protocol approved by the Partners HealthCare Institutional Review Board. All study participants provided written informed consents. Participants carrying pathogenic variants in other PD-associated genes (*SNCA*, *LRRK2*, *VPS35*, *PRKN*, *PINK1* and *PARK7*) were excluded.

### The Luxembourg Parkinson’s study

For independent replication, we used 653 PD patients and 767 healthy controls from LuxPark^21,22^, a longitudinal monocentric study within the framework of the NCER-PD (National Center for Excellence in Research in PD). All NCER-PD participants provided written informed consent, and the study was approved by the National Research Ethics Committee (CNER Ref: 201407/13). Genotyping was carried out using the NeuroChip platform^23^, while *GBA1* variants were identified using the *GBA1*-targeted PacBio sequencing method^24^. QC for the genotyping data has been previously described^22^. Participants carrying pathogenic single nucleotide variants (SNVs) or copy number variants (CNVs) in PD-associated genes (*SNCA*, *LRRK2*, *VPS35*, *PRKN*, *PINK1* and *PARK7*) were excluded^22^. Additionally, we excluded individuals harboring variants of uncertain significance (VUS) or synonymous variants in the *GBA1* gene. All subjects gave written informed consent. The study was approved by the National Research Ethics Committee (CNER Ref: 201407/13).

### Classification of *GBA1* variants

We classified *GBA1* variants based on their pathogenicity in relation to PD and GD, categorizing them as risk, mild, or severe, following the classification of Höglinger and colleagues^4^, without further stratification by allele count. Variants identified as pathogenic for GD were classified as either mild or severe for PD, with severe variants exhibiting an odds ratio (OR) of 10–15 for developing PD, while mild variants had an OR of ≤5^25,26^. Common variants not considered pathogenic for GD but known to increase PD risk (e.g., p.E365K, p.T408M) were categorized as risk variants. Frameshift and nonsense variants in *GBA1* were classified as severe. Variants not classified by Höglinger et al. were further categorized (as severe, mild, or risk) according to the classification of Parlar and colleagues^2^ using the online *GBA1* variant browser (https://pdgenetics.shinyapps.io/gba1browser/). Notably, carriers were identified by the presence of variants, regardless of zygosity. In the LuxPark cohort, two PD patients were found to be homozygous for the E365K risk variant.

### Polygenic risk score

To generate the PRS, we used a list of 44 SNPs available from a previous meta-analysis of PD^27^. These SNPs were identified in independent datasets, minimizing bias from UK Biobank sample overlap with the Nalls et al. 2019 GWAS^8^. Variants within the *GBA1* (rs35749011) and *LRRK2* (rs76904798) loci were excluded from the PRS calculation, resulting in a final set of 42 SNPs used for PRS calculation. We computed PRS using PRSice2^28^, where SNPs genotypes were treated as allele count (0, 1, 2) and weighted by their respective effect sizes, while accounting for allele-flipping. The analysis was performed using the ‘–no-regress’ and ‘–no-clumping’ options alongside default parameters.

## Statistical analysis

To investigate the association between PRS, *GBA1*_PVs_ carrier status, and PD risk, we performed logistic regression and Cox proportional hazards regression with disease occurrence as the outcome variable. Participants were stratified based on their PRS and *GBA1*_PVs_ carrier status. Initially, individuals were categorized into equal tertiles based on their PRS distribution. Those in the lowest tertile were assigned to the low-PRS group, those in the highest tertile group to the high-PRS group, and those in the middle tertile to the intermediate-PRS group.

For both cohorts, ORs were estimated using a logistic regression model, conditioning on covariates such as sex, age at assessment, and the first four principal components. In the UK Biobank dataset, we used the principal components (PC1–4) provided by the UK Biobank, whereas for the Luxembourg Parkinson’s Study, we calculated PCs using PLINK. We applied Cox proportional hazard regression model to evaluate time-to-event data and estimate cumulative incidence risk of PD. Afterwards, we additionally incorporated interactions between *GBA1*_PVs_ status and PRS by introducing an interaction term within the logistic regression model. Finally, we further categorized the *GBA1*_PVs_ carriers’ participants into two groups: (1) mild and severe *GBA1*_PVs_ carriers and (2) risk *GBA1*_PVs_ carriers. This classification allowed to evaluate the association between different levels of *GBA1*_PVs_ severity and the risk of developing PD. The reference category is given by non-carriers with intermediate-PRS. We used R v4.2.2 for all statistical analyses.

## Supplementary information

supplementary_material_revision_02052025

## Data Availability

Data used to prepare this article were obtained from UKB, and the National Center of Excellence in Research: Early diagnosis and stratification of Parkinson’s Disease (NCER-PD or the LuxPark https://www.parkinson.lu). Restrictions apply to the availability of these data for UKB, which were used under license for the current study (Project ID: 73507). Patient data used in the preparation of this manuscript were obtained from the National Center of Excellence in Research on Parkinson’s Disease (NCER-PD). NCER-PD datasets are not publicly available, as they are linked to the Luxembourg Parkinson’s Study and its internal regulations. The NCER-PD Consortium is willing to share its available data. Its access policy was devised based on the study ethics documents, including the informed consent form, as approved by the national ethics committee. Requests to access datasets should be directed to the Data and Sample Access Committee via email: request.ncer-pd@uni.lu.
