# Supplementary material for: Penetrance of Parkinson’s disease in GBA1 carriers depends on variant severity and polygenic background
Source: NPJ Parkinsons Dis. 2025 Jun 12;11:162. doi: 10.1038/s41531-025-00997-y (PMC12162886; doi:10.1038/s41531-025-00997-y)
Supplement: Supplementary file 1 — supplementary_material_revision_02052025 [file 41531_2025_997_MOESM1_ESM.pdf]

Supplemental Table 1: Cumulative incidence of Parkinson's disease (PD) stratified by *GBA1* carrier status and polygenic risk scores categories in UK Biobank.

GBA1 Carriers	PRS	Cumulative risk %<br>70 years (95% CI)
GBA1 Carrier	High	12.8% (2.7)
	Intermediate	7.97% (1.6)
	Low	5.84% (1.2)
Non-carrier	High	7.24% (1.51)
	Intermediate	4.33% (0.91)
	Low	3.60% (0.74)

Supplemental Table 2: SNPs showing nominally significant differences between PD cases vs controls in *GBA1* carriers and non-carriers.

**Study**	**Group**	**rs ID**	**Fisher-exact test p-value**	**FDR-corrected p-value**
UK Biobank	Non-carriers	rs11158026	2.20e-06	8.58e-05
		rs356182	1.49e-05	2.90e-04
		rs1474055	3.34e-04	3.31e-03
		rs11724635	3.40e-04	3.31e-03
		rs17649553	1.03e-03	6.89e-03
		rs34311866	1.06e-03	6.89e-03
		rs14235	2.92e-03	1.63e-02
		rs117896735	9.55e-03	4.55e-02
	GBA1-carriers	rs1474055	0.0112	0.179
		rs601999	0.0118	0.179
		rs356182	0.0138	0.179
		rs6430538	0.0197	0.192
LuxPark	Non-carriers	rs34311866	0.001152859	0.04726723
	GBA1-carriers	rs1555399	0.003984293	0.1633560

Supplemental Table 3: Variability in PD cumulative incidence among *GBA1* carriers across studies.

Study group	Cumulative risk %		Citations
	60 years	70 years	
GBA1 Carriers	5		McNeil et al, 2012[1]
Familial cohort	13.7	21.4	Anheim et al, 2012[2]
GBA1 Carriers	1.0 ± 1.4		Rana et al, 2013[3]
GBA1 Carriers	1.5 ± 0.98	5.2 ± 1.96	Alcalay et al, 2014[4]
Patients with GD	4.7 ± 3.33	9.1 ± 6.07	Alcalay et al, 2014[4]
GBA1 Carriers	10	16	Balestrino et al, 2020[5]
GBA1 Carriers (UKB)	1.7 ± 0.9	10.1 ± 2.1	Current study

Supplemental Figure 1: Parkinson's disease risk over time stratified by *GBA1* carrier status and polygenic risk categories in LuxPARK

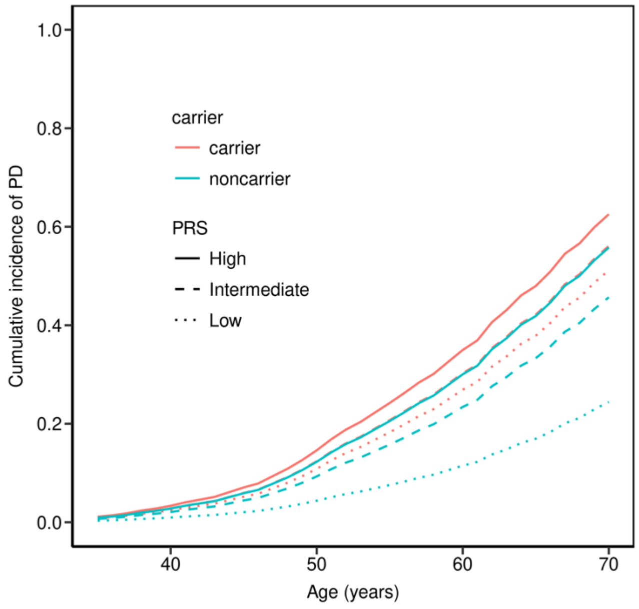

Supplemental Figure 2: PD risk stratified by polygenic risk scores categories and the status and severity of rare pathogenic *GBA1* variants in UK Biobank (A), LuxPark (B).

A)

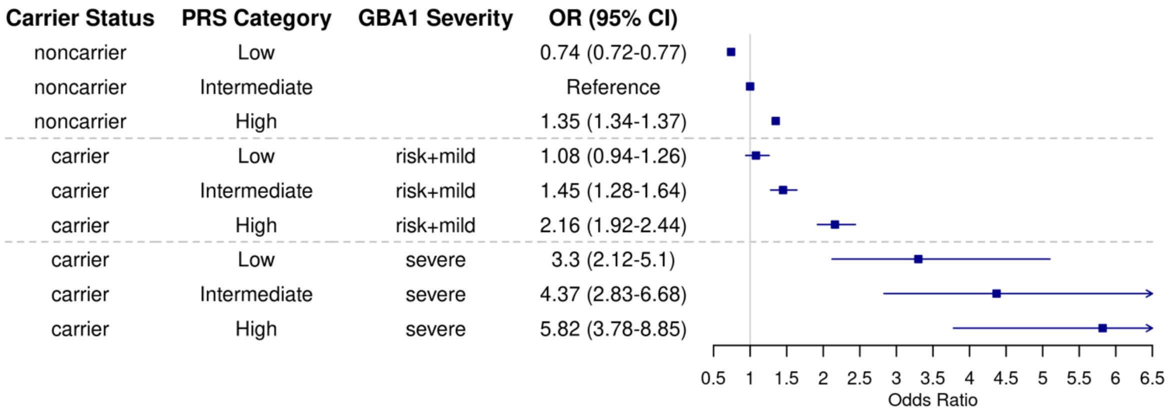

B)

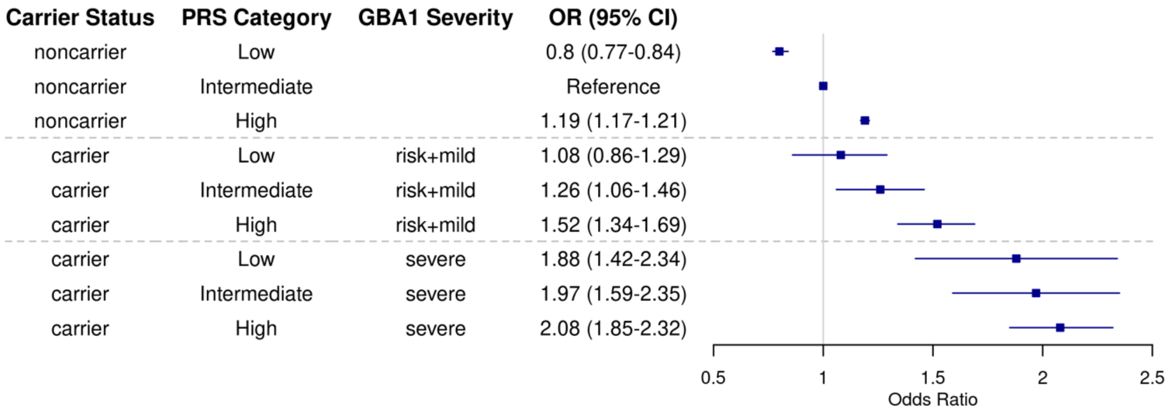

References

1. McNeill, A., Duran, R., Hughes, D. A., Mehta, A. & Schapira, A. H. V. A clinical and family history study of Parkinson's disease in heterozygous glucocerebrosidase mutation carriers. *J. Neurol. Neurosurg. Psychiatry* **83**, 853–854 (2012).

2. Anheim, M. *et al.* Penetrance of Parkinson disease in glucocerebrosidase gene mutation carriers. *Neurology* **78**, 417–420 (2012).
3. Rana, H. Q., Balwani, M., Bier, L. & Alcalay, R. N. Age-specific Parkinson disease risk in GBA mutation carriers: information for genetic counseling. *Genet. Med.* **15**, 146–149 (2013).
4. Alcalay, R. N. *et al.* Comparison of Parkinson Risk in Ashkenazi Jewish Patients With Gaucher Disease and GBA Heterozygotes. *JAMA Neurol.* **71**, 752–757 (2014).
5. Balestrino, R. *et al.* Penetrance of Glucocerebrosidase () Mutations in Parkinson's Disease: A Kin Cohort Study. *Mov. Disord.* **35**, 2111–2114 (2020).
